# Integration of the *ICD-11* and *DSM-5* Dimensional Systems for Personality Disorders Into a Unified Taxonomy With Non-overlapping Traits

**DOI:** 10.3389/fpsyt.2021.591934

**Published:** 2021-04-06

**Authors:** Fernando Gutiérrez, Josep M. Peri, Miguel Gárriz, Gemma Vall, Estela Arqué, Laura Ruiz, Jaume Condomines, Natalia Calvo, Marc Ferrer, Bárbara Sureda

**Affiliations:** ^1^Institute of Neuroscience, Hospital Clinic, Barcelona, Spain; ^2^Institut d'Investigacións Biomèdiques August Pi Sunyer (IDIBAPS), Barcelona, Spain; ^3^Neuropsychiatry and Drug Addiction Institute, Parc de Salut Mar, Barcelona, Spain; ^4^Department of Psychiatry, Mental Health, and Addiction, GSS–Hospital Santa Maria, Lleida, Spain; ^5^Biomedical Research Institute, Lleida, Spain; ^6^La Coma Therapeutic Community, ATRA Group, Barcelona, Spain; ^7^Psychiatry Department, Vall d'Hebron University Hospital, Centro de Investigación Biomédica en Red de Salud Mental (CIBERSAM), Barcelona, Spain; ^8^Psychiatry and Legal Medicine Department, Autonomous University of Barcelona, Barcelona, Spain

**Keywords:** *ICD-11*, *DSM-5*, personality disorders, discriminant validity, general factor

## Abstract

The promise of replacing the diagnostic categories of personality disorder with a better-grounded system has been only partially met. We still need to understand whether our main dimensional taxonomies, those of the *International Classification of Diseases*, 11th Revision (*ICD-11*) and the *Diagnostic and Statistical Manual of Mental Disorders*, Fifth Edition (*DSM-5*), are the same or different, and elucidate whether a unified structure is possible. We also need truly independent pathological domains, as they have shown unacceptable overlap so far. To inquire into these points, the Personality Inventory for *DSM-5* (PID-5) and the Personality Inventory for *ICD-11* (PiCD) were administered to 677 outpatients. Disattenuated correlation coefficients between 0.84 and 0.93 revealed that both systems share four analogous traits: negative affectivity, detachment, dissociality/antagonism, and disinhibition. These traits proved scalar equivalence too, such that scores in the two questionnaires are roughly interchangeable. These four domains plus psychoticism formed a theoretically consistent and well-fitted five-factor structure, but they overlapped considerably, thereby reducing discriminant validity. Only after the extraction of a general personality disorder factor (g-PD) through bifactor analysis, we could attain a comprehensive model bearing mutually independent traits.

## Introduction

Despite being increasingly close to a scientifically based personality disorder (PD) classification, we still do not have a unique, generally accepted, and unproblematic dimensional substitute for the traditional categories (1, 2). An important step in this direction would entail collating the different dimensional models currently at our disposal, each of them somewhat different from the others, such that we can elucidate which personality domains are common, which differ, and how we can arrange all them in the best possible way to form a comprehensive nosology. Agreement is particularly important regarding the *ICD-11* model (3) and the *DSM-5* alternative model for PD (AMPD) (4), both because they are our two main dimensional classificatory systems, and because they themselves have been attempts to unify the pre-existing trait taxonomies (5, 6).

As the *ICD-11* will be the authoritative diagnostic taxonomy since 2022, it is the natural framework against which other models should be compared. Attempts have been made to capture the *ICD-11* domains from the more broadly studied Personality Inventory for *DSM-5* (PID-5) (7, 8), and the resulting factors confirmed the presumption that both models are commensurate. However, these studies did not take psychoticism into account, as it does not form part of the *ICD-11* classification, and their design did not allow testing a common structure for the AMPD and the *ICD-11*. Given the clinical relevance of psychoticism (or schizotypy) (9), such a comprehensive model would be worthwhile. Six further studies have compared the PID-5 with instruments directly designed to capture the *ICD-11* domains, either the Personality Inventory for *ICD-11 (*PiCD) (10) or the Five-Factor Personality Inventory for *ICD-11* (FFiCD) (11). Convergence between both systems was found to be good for four of the domains: negative affectivity (*r* = 0.75–0.86), detachment (0.60–0.80), dissociality/antagonism (0.67–0.81), and disinhibition (0.73–0.89) (10–15). Even so, results need to be replicated and extended in several respects.

These studies have not examined face-to-face clinical samples, which are the natural target of a diagnostic taxonomy. Four studies used internet-based self-declared patients (10, 11, 13, 14) and the other two were conducted with community subjects (12, 15). On the other hand, common structure has been analyzed at the PID-5 domain level, with one sole exception (14). This reduces the number of indicators per construct to unacceptable levels, then precluding psychoticism to form a separate factor and leaving little room for testing alternative solutions (16). Furthermore, most factor analyses have been conducted in conjunction with other models, either the Big Five or those of Zuckerman, Livesley, or Clark (11–13, 15). Even if insightful, this approach may reshape the resulting structure, such that the shared configuration of the two official classifications is obscured. This has been specially the case with the anankastia and psychoticism domains. The well-stablished bipolar factor with disinhibition and anankastia at opposite extremes has been replicated in some studies (10, 14, 15), but has split in different ways in others, depending on the accompanying model (12, 13). Concerning psychoticism, it has been sometimes excluded from analysis (11), while others has formed an independent factor (10, 14), and still others has loaded into the dissociality, disinhibition, or negative affectivity factors (12, 13, 15).

Importantly, good convergent validity for the four analogous domains has invariably been coupled with poor discriminant validity, with non-corresponding correlations averaging 0.29 in the community and 0.40 in patients (10–15). Thus, the promise that blurred boundaries between traditional diagnostic categories would be fixed by a dimensional system has not be met. In this respect, it has been proposed that a general factor of personality disorder (g-PD) may permeate every dimension of personality pathology (17), producing undue overlap. A suitable approach to examine this issue is bifactor analysis (18). It involves extracting an all-inclusive factor that captures variance of all traits (the general factor) and that is orthogonal to a variable number of other factors gathering the remaining covariance between narrower groups of traits (specific factors). The only attempt so far has indeed provided a clearer factor structure (11). However, the exclusion of psychoticism in accordance with the *ICD-11* model precluded the appearance of a fifth substantial factor in this study, and model fit and correlations between the resulting factors were not reported. Nor has exploratory structural equation modeling (ESEM) (19, 20) been hitherto applied to the common structure of the PiCD and the PID-5, though it has been to *DSM*-based algorithms (7, 8). This method is strongly recommended instead of the more usual exploratory (EFA) and confirmatory (CFA) approaches, as it relaxes some unreal constraints such as zero cross-loadings, which are unlikely to be fulfilled by personality structures (20). Therefore, it can adjust previously obtained exploratory structures that are rarely supported by CFA (21).

Finally, no studies have tested the direct equivalence of the PiCD and PID-5 scores beyond Pearson correlations. On the one hand, disattenuated correlations, which discount measurement error, will be closer to the true associations between domains (22). On the other, scalar equivalence between the two instruments is untested, that is, whether a certain score on the PiCD would correspond with a similar score on the PID-5, qualifying them as interchangeable (23).

Due to its recent publication, there is a paucity of data on the *ICD-11* classification of PD. The present study sought to replicate and extend the relationships between our main classificatory systems for PD, the *ICD-11* and the AMPD. Specifically, we examined in a sample of 677 outpatients, the extent to which both systems are similar or different, and how a unified and maximally comprehensive taxonomic system, comprising non-overlapping pathological traits, should ultimately look like.

## Materials and Methods

### Subjects

The PID-5-SF and the PiCD were administered to 677 outpatients, 65.7% women, aged 13–81 years (*M* = 38.4, *SD* = 13.2), consecutively referred for assessment or treatment to five different mental health or drug addiction units in Catalonia, Spain. Patients were clinically diagnosed at their respective centers according to the *DSM-5* (4), with 18.6% of them presenting a phobic or other anxiety disorder, 18.6% mixed anxious and depressive symptoms, 11.5% a mild to moderate depressive disorder, 8.1% other affective disorders (dysthymia, cyclothymia, bipolar disorder), 15.4% drug abuse disorders, and 15.9% other disorders—eating, obsessive-compulsive, impulse control disorders—each with a frequency of under 5%.

### Instruments

The Personality Inventory for *ICD-11* (PiCD) (10) is a 60-item self-report assessing the five-dimensional personality disorder classification of the *ICD-11* (3): negative affectivity, detachment, dissociality, disinhibition, and anankastia. Each domain has 12 items rated from 1 (*strongly disagree*) to 5 (*strongly agree*). The Personality Inventory for *DSM-5* Short Form (PID-5-SF) (24, 25) is a 100-item self-report measuring the 25 facets and five domains of the alternative model of the *DSM-5* (4): negative affectivity, detachment, antagonism, disinhibition, and psychoticism. It is scored on a scale ranging from 0 (*strongly disagree*) to 3 (*strongly agree*) and has shown psychometric properties similar to the 220-item PID-5, on which it is based (26). The questionnaires were administered in their Spanish versions (27–29) as part of a routine personality evaluation by an experienced, doctoral-level clinical psychologist. They were delivered in the same order (PID-5 and then PiCD), in pencil-and-paper format, following an interview in which procedures were explained.

## Results

Summary statistics for the PID-5 and the PiCD are shown in Supplementary Tables 1–8. Normative T-scores were taken from the Spanish validation studies (27, 28). We first examined by means of Pearson's correlations the extent to which the PiCD and PID-5-SF domains measure the same constructs and can be considered equivalent. In order to count out measurement error, coefficients were disattenuated, that is, divided by the square root of the product of the alpha reliabilities of each pair of variables $r_{c}=\;\frac{r_{xy}}{\sqrt{r_{xx}\times r_{yy}}}$ (22). Table 1 confirms a clear correspondence between four of the domains —negative affectivity, detachment, dissociality/antagonism, and disinhibition—, with r coefficients from 0.72 to 0.80, and r_c_ from 0.84 to 0.93.

**Table 1:** Pearson's (lower triangle) and disattenuated correlations (upper triangle) between the PiCD and PID-5 domains (*n* = 677).

	**PiCD**						**PID-5**					
	**Neg. affectivity**	**Detachment**	**Dissociality**	**Disinhibition**	**Anankastia**	**Total score**	**Neg. affectivity**	**Detachment**	**Antagonism**	**Disinhibition**	**Psychoticism**	**Total score**
**PiCD**												
Neg. affectivity	—	0.35	0.34	0.51	0.11	**0.87**	**0.87**	0.39	0.48	0.53	0.07	**0.82**
Detachment	0.29	—	0.29	0.35	0.14	**0.82**	0.39	**0.84**	0.34	0.44	−0.01	**0.71**
Dissociality	0.28	0.24	—	**0.61**	−0.13	**0.80**	0.25	0.15	**0.86**	0.53	−0.15	0.56
Disinhibition	0.43	0.29	0.50	—	−0.57	**0.75**	0.54	0.36	**0.63**	**0.93**	−0.47	**0.71**
Anankastia	0.09	0.11	−0.10	−0.46	—	0.24	0.54	0.47	0.52	0.52	−0.06	**0.69**
Total Score	**0.73**	**0.68**	**0.65**	**0.63**	0.19	—	**0.72**	0.54	**0.68**	**0.72**	−0.12	**0.90**
**PID-5**												
Neg. affectivity	**0.77**	0.34	0.22	0.47	0.46	**0.65**	—	0.53	0.43	**0.68**	**0.69**	**0.93**
Detachment	0.34	**0.72**	0.13	0.31	0.40	0.48	0.48	—	0.25	0.51	0.56	**0.72**
Antagonism	0.41	0.29	**0.74**	0.53	0.44	**0.60**	0.40	0.23	—	**0.60**	0.43	**0.69**
Disinhibition	0.47	0.38	0.47	**0.80**	0.45	**0.65**	**0.62**	0.45	0.54	—	**0.64**	**0.88**
Psychoticism	0.06	−0.01	−0.13	−0.39	−0.05	−0.10	**0.62**	0.50	0.39	0.57	—	**0.84**
Total Score	**0.72**	**0.61**	0.49	**0.61**	0.59	**0.80**	**0.88**	**0.67**	**0.65**	**0.81**	**0.77**	—

*PID-5, Personality Inventory for the DSM-5—Short Form; PiCD, Personality Inventory for ICD-11. Correlations are significant at p < 0.05 from r = 0.07 and at p < 0.01 from r = 0.10. Coefficients ≥ 0.60 are in bold type*.

Assuming that there is not a biunivocal correspondence between the two models, we also examined if each domain in one model can be explained by a linear combination of several domains in the other. Although multiple regression analyses confirmed that four out of five factors in each model were reasonably well-explained by the other model, additional predictors did not bring an improvement with respect to correlations: R coefficients ranged from 0.72 to 0.80 for the four corresponding PiCD domains predicted by the PID-5 (mean *R* = 0.77, *R*^2^ = 0.53) and from 0.75 to 0.84 for the four corresponding PID-5 domains predicted by the PiCD (mean *R* = 0.79, *R*^2^ = 0.57; Supplementary Table 2). Coefficients for PiCD anankastia (*R* = 0.55 using domains and 0.70 using facets) and PID psychoticism (0.62) were not as high as for the other domains, though anankastia was clearly predicted by disinhibition and negative affectivity. Both models were about equally comprehensive of the other's domains, but the inclusion of the PID-5 facets rather than domains improved prediction to *R* = 0.81 (*R*^2^ = 0.62) on average.

**Figure 1:**
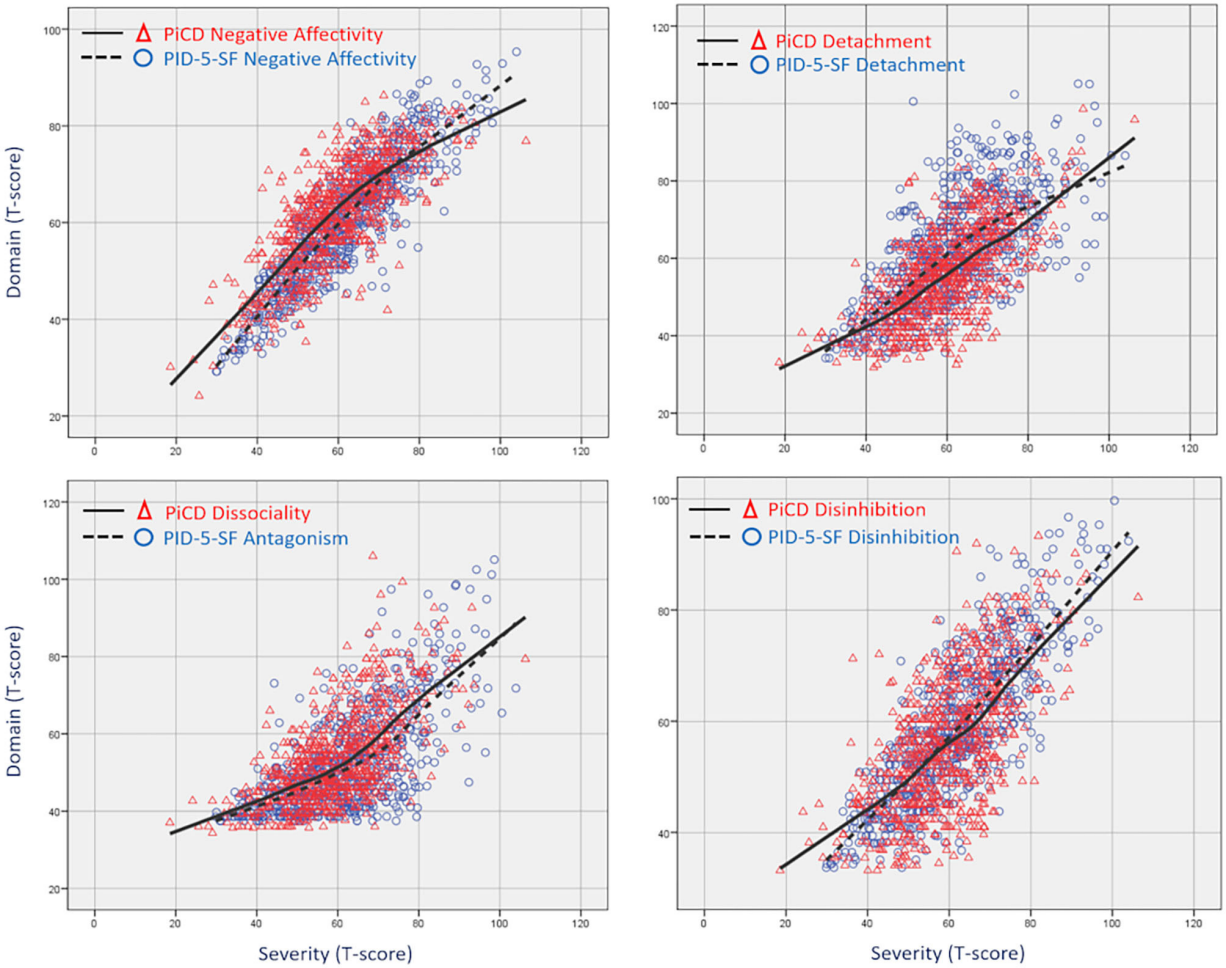
Loess curves showing similar scores for the PiCD and the PID-5 domains as severity increases (*n* = 677). For each domain, severity is the total score of its corresponding questionnaire: PiCD or PID-5.

In order to study scalar equivalence, we compared T-scores between the two questionnaires. We sought to know whether the corresponding domains of each questionnaire scored similarly, and therefore they are interchangeable. To this end, we used the two one-sided tests (TOST) procedure for paired data implemented in the TOSTER package for R (23). Rather than testing mean differences, TOST more appropriately checks for mean equivalences, that is, whether patients scoring at a given level in one domain fall within predefined lower and upper bounds in the other model's corresponding domain. With bounds set at ΔT < ±5, only PID-5 detachment failed to show significant equivalence (*t* = 2.93, *p* = 0.998), whereas negative affectivity (*t* = 12.00, *p* < 0.001), antagonism (*t* = 11.81, *p* < 0.001), and disinhibition (*t* = −7.33, *p* < 0.001) proved to be equivalent between models. Similar results were found when this approach was applied in four different levels of severity, defined as the total score of each questionnaire expressed in T-scores. Whereas PID-5 negative affect, antagonism, and disinhibition differed little, PID-5 detachment scored between half and three quarters of a standard deviation higher all along the gradient from *T* = 50 to 90 (Supplementary Table 3). These results can be illustrated through a locally weighted scatterplot smoothing (LOESS) graph with severity in the *x*-axis and the four domains in the *y*-axes (Figure 1).

Whereas Table 1 confirms the convergence between the PiCD and PID-5 corresponding domains, it also reveals considerable overlap between non-corresponding domains (mean *r* = 0.36). We undertook a series of factor analyses to examine whether the PiCD and the PID-5 can share a common structure formed by sufficiently differentiated constructs. Joint exploratory factor analyses (EFA) were conducted at the domain level first, using maximum likelihood extraction (ML) and oblique Geomin rotation, as factors were expected to correlate. Parallel analysis and Bayesian information criterion (BIC) suggested retaining four factors (Supplementary Figure 1), and Velicer's MAP suggested one. The 4-factor solution reproduced four of the expected constructs—negative affectivity, detachment, dissocial/antagonism, and disinhibition/anankastia, whereas extracting a fifth factor failed to recover psychoticism (Supplementary Tables 4, 5). This approach was unsatisfactory in other respects. Congruence with alternative extractions—weighted and unweighted least squares and principal axis factoring—and with Oblimin, Promax, and Equamax rotations was acceptable for the 4-factor solution (Tucker's *Φ* between 0.91 and 1.00, mean 0.98) but failed for the 5-factor solution (*Φ* between 0.55 and 1.00, mean 0.88), suggesting an unstable and hardly replicable structure (30). Moreover, factors were highly intercorrelated (mean *r* = 0.38 in the 4-factor and 0.46 in the 5-factor solutions). Using orthogonal instead of oblique rotations fixed this problem at the cost of unduly increasing cross-loadings.

Hence, we jointly analyzed the 25 PID-5 facets and five PiCD domains, expecting that more indicators would produce a clearer structure. This was accomplished to some extent. Parallel analysis, Velicer's MAP, and BIC coincided in suggesting five factors. A 5-factor solution showed excellent congruence with alternative extraction and rotation methods (*Φ* between 0.96 and 1.00, mean 0.99) and reproduced well the original constructs (Supplementary Table 6): negative affect (*r* = 0.86 with PiCD and 0.95 with PID-5), detachment (0.86 and 0.85), dissocial/antagonism (0.84 and 0.96) and disinhibition/anankastia (0.75 and 0.62 with disinhibition, −0.83 with anankastia). An ESEM analysis was then performed in lavaan R package (31) to evaluate model fit. To this end, factor variances were fixed, and all cross-loadings estimated through robust ML with the EFA loadings as starting points. Fit was within acceptable limits: χ^2^ = 1302.06, *df* = 420, *p* < 0.001, CFI = 0.924, TLI = 0.921, RMSEA = 0.056, and SRMR = 0.028. The 4-factor solution also showed good congruence between methods, but fused the negative affectivity and psychoticism factors together (Supplementary Table 7) and showed lower fit: χ^2^ = 1769.21, *df* = 425, *p* < 0.001, CFI = 0.884, TLI = 0.882, RMSEA = 0.068, and SRMR = 0.039. Both solutions partially improved discriminant validity, with factor intercorrelations averaging 0.22 in the 5-factor and 0.24 in the 4-factor solution (Supplementary Table 6).

Finally, we tested the existence of a general factor of personality disorder (g-PD) underlying all traits, which would explain the considerable overlap between them. PiCD domains and PID-5 facets were subjected to a purely exploratory bifactor analysis in FACTOR 10.10 (32, 33), retaining 4 + 1 and 5 + 1 factors. Loadings were then targeted in ESEM analysis as above. Fit was slightly better for the 5 + 1 solution (χ^2^ = 1127.09, *df* = 419, *p* < 0.001, CFI = 0.939, TLI = 0.937, RMSEA = 0.050, and SRMR = 0.025) than for the 4 + 1 solution (χ^2^ = 1256.38, *df* = 390, *p* < 0.001, CFI = 0.923, TLI = 0.920, RMSEA = 0.057, and SRMR = 0.028). In the former (Table 2), the g-PD factor received substantial and homogeneous contributions from all traits except anankastia and rigid perfectionism, which qualifies it as a genuine general factor. Specific factors still correlated in the range 0.56–0.74 with their corresponding PiCD and PID-5 domains. Discriminant capacity was improved, with quasi-orthogonal factors (mean *r* = 0.12) and low correlations (<0.30) with non-corresponding domains. Only anankastia and dissociality showed intrusions into negative affectivity and disinhibition, respectively. Similar remarks can be made regarding the 4+1 model, whose factor intercorrelations averaged 0.08, but in which psychoticism was assimilated by the g-PD (Supplementary Table 8).

**Table 2:** ESEM 5+1-factor solution for the PiCD domains and PID-5 facets targeted to the purely exploratory bifactor analysis (*n* = 677).

	**g-PD**	**Neg. affectivity**	**Detachment**	**Dissocial**	**Disinhibition**	**Psychoticism**
**Factor solution**						
PiCD negative affectivity	**0.44**	**0.69**	−0.03	−0.06	0.26	0.06
PiCD detachment	**0.47**	0.03	**0.69**	−0.15	0.04	0.05
PiCD dissociality	**0.51**	0.02	0.00	**0.60**	0.21	0.04
PiCD disinhibition	**0.69**	−0.04	−0.05	−0.05	**0.54**	−0.06
PiCD anankastia	−0.23	**0.40**	0.19	0.25	**−0.52**	0.06
PID-5 anxiousness	**0.42**	**0.68**	−0.04	−0.03	0.05	0.02
PID-5 rigid perfectionism	0.25	**0.55**	0.07	0.28	−0.12	0.18
PID-5 emotional lability	**0.36**	**0.55**	−0.25	−0.11	0.29	0.11
PID-5 separation insecurity	**0.37**	**0.44**	**−0.31**	0.04	0.03	−0.04
PID-5 perseveration	**0.54**	**0.42**	−0.02	−0.01	0.19	−0.04
PID-5 submissiveness	**0.39**	**0.35**	−0.03	−0.06	−0.10	−0.17
PID-5 suspiciousness	**0.59**	**0.32**	0.05	0.13	0.08	0.21
PID-5 depressiveness	**0.62**	**0.32**	0.22	−0.23	0.21	0.10
PID-5 restricted affect	**0.43**	−0.19	**0.62**	0.03	−0.05	0.00
PID-5 withdrawal	**0.46**	0.14	**0.60**	−0.19	0.07	0.08
PID-5 intimacy avoidance	**0.30**	−0.16	**0.43**	−0.19	0.14	0.15
PID-5 anhedonia	**0.63**	**0.31**	**0.32**	−0.16	0.15	−0.07
PID-5 manipulativeness	**0.49**	−0.08	−0.15	**0.68**	0.02	−0.10
PID-5 deceitfulness	**0.66**	−0.04	−0.16	**0.56**	−0.02	−0.19
PID-5 attention seeking	**0.39**	0.10	**−0.39**	**0.55**	−0.01	−0.11
PID-5 grandiosity	**0.39**	0.09	−0.06	**0.54**	−0.14	−0.03
PID-5 callousness	**0.55**	−0.17	0.11	**0.36**	0.08	−0.02
PID-5 impulsivity	**0.55**	0.15	−0.17	−0.01	**0.54**	0.07
PID-5 hostility	**0.48**	**0.34**	0.10	0.12	**0.37**	0.01
PID-5 risk taking	**0.49**	−0.14	−0.06	0.20	**0.31**	0.27
PID-5 unusual beliefs	**0.55**	−0.01	−0.02	−0.03	0.06	**0.62**
PID-5 perceptive distortion	**0.61**	0.03	−0.07	−0.18	−0.12	**0.61**
PID-5 eccentricity	**0.57**	0.17	0.15	−0.05	0.16	**0.33**
PID-5 irresponsibility	**0.69**	−0.09	0.01	0.11	0.26	−0.17
PID-5 distractibility	**0.55**	0.24	0.04	−0.21	0.24	−0.06
**Correlations with domains**						
PiCD negative affectivity	**0.47**	**0.74**	0.03	−0.03	0.26	0.16
PiCD detachment	**0.50**	0.13	**0.76**	−0.03	−0.04	0.10
PiCD dissociality	**0.55**	−0.02	0.10	**0.73**	**0.40**	0.25
PiCD disinhibition	**0.73**	−0.08	−0.12	0.04	**0.66**	−0.07
PiCD anankastia	−0.25	**0.49**	**0.36**	0.19	**−0.61**	0.20
PID-5 negative affectivity	**0.69**	**0.70**	0.05	0.06	0.18	0.22
PID-5 detachment	**0.65**	0.14	**0.69**	−0.05	0.03	0.09
PID-5 antagonism	**0.68**	−0.10	−0.11	**0.72**	0.15	0.04
PID-5 disinhibition	**0.81**	0.03	−0.10	0.10	**0.56**	0.08
PID-5 psychoticism	**0.73**	0.18	0.10	0.08	0.06	**0.68**
**Factor intercorrelations**						
g-PD	–					
Neg. affectivity	0.06	**–**				
Detachment	0.07	0.11	–			
Dissociality	0.11	−0.11	0.09	–		
Disinhitibition	0.16	−0.12	−0.20	0.11	–	
Psychoticism	0.10	0.14	0.08	0.22	−0.06	–

*PID-5, Personality Inventory for the DSM-5—Short Form; PiCD, Personality Inventory for ICD-11. Correlations and factor loadings ≥ 0.30 are in bold type*.

## Discussion

We examined the relationships between the *ICD-11* and *DSM-5* systems for PD as measured through the PiCD and PID-5-SF questionnaires in 677 outpatients. We found that both systems share four basic constructs of personality pathology: negative affectivity, detachment, dissociality/antagonism, and disinhibition/anankastia. Once measurement error is taken into account, correlations range from 0.84 to 0.93 between these factors. Two domains, anankastia and psychoticism, maintain more complex relationships between the two models. However, because of their clinical relevance (9, 34), they must be kept in the nosology, which legitimates the attempt to integrate both models instead of selecting one over the other. Indeed, our 5-factor and 5+1-factor models capture anankastia (*R* = 0.84 and 0.86, respectively) and psychoticism (both *R* = 0.96) at once, while the *ICD-11* and the AMPD taken individually cannot. Furthermore, the PiCD and the PID-5-SF use the same metric, such that scores are approximately equivalent between them. A relative exception is detachment, for which the PID-5 scored about half a standard deviation higher than the PiCD. Supplementary Table 1 suggests that this may be attributed to high levels of anhedonia in our patients, a feature which is less prominent in the PiCD.

We confirmed the prior finding that the extraction of a g-PD fixes the otherwise serious problems of blurred boundaries between domains (11). Under factor analysis as usual, constructs overlap extensively with each other—as the original domains also do—, thus decreasing discriminant validity. Contrarily, bifactor modeling produces an unequivocal general factor that uniformly underlies every trait except anankastia, as well as five factors that correspond with the four *ICD-11* domains plus psychoticism. The latter is one major difference with Oltmanns and Widiger (11), who circumscribed the analysis to the four-factor *ICD-11* framework. Furthermore, the 5 + 1 model fits the data well in ESEM and produces quasi-orthogonal constructs. Although giving preference to a bifactor model on the sole basis of good fit is discouraged (21), this is not our case: Obtaining sufficiently differentiated traits is a worthwhile achievement in itself, and the empirical and theoretical bases supporting the existence of a general factor underlying PD are on the rise (17).

This does not imply an agreement on the interpretation of the g-PD. This construct has been understood quite heterogeneously as a response bias, as another name for neuroticism or borderline organization, as a general vulnerability to psychopathology, or as the conjoint of maladaptive consequences shared by all disorders (17). Either way, it is a plausible explanation for the well-established fact that all personality disorders—in fact, all mental disorders—correlate with each other (35). For example, the psychoticism factor correlates 0.51 on average with the non-corresponding PID-5 domains in our study (Supplementary Table 6), but only 0.11 after the g-PD has been factorially isolated (Table 2). Thus, the g-PD may partly explain why psychoticism maintains unspecific associations with all other domains and with the Big Five, including high neuroticism and low agreeableness and conscientiousness (36), and why it predicts psychosis but also other psychopathology (37, 38). At the facet level, this effect seems more pronounced for eccentricity than for perceptual dysregulation and unusual beliefs (Table 2), which are also better able to predict psychosis (37, 39). Our findings are in line with the proposal that pathological traits are a mixture of normal-range traits plus unspecific personality dysfunction (40), but leave unsolved how to interpret pathological traits once the maladaptation component has been removed. Not less important, the *ICD-11* and AMPD systems have proposed separate constructs for personality traits and severity/dysfunction, but these have been found to be ultimately redundant (41, 42). It is a pending question if the g-PD might represent this common component of dysfunction, and if it could explain the overlap between dysfunction and traits.

A weakness of this study is that sample size did not allow to have exploratory and confirmatory subsamples, so our factor solutions may partially capitalize on chance. Furthermore, we have focused in only a part of the *ICD-11* and AMPD systems, namely trait models. Although the inclusion of additional components—the borderline specifier in the *ICD-11*, six diagnostic categories in the AMPD, and the severity/dysfunction measures of each system—would have offered a more complete picture, it would also have led to excessive complexity for one sole study. Furthermore, using the FFiCD (11) instead of the PiCD would have enriched our facet-level analyses, but this instrument was not yet been published when our study began.

In sum, we have found considerable agreement between the two main dimensional taxonomies for PD, the *ICD-11* and the AMPD. As they have been developed independently, this can be regarded as a strong validation for both. Interestingly, divergences between models can be settled if we integrate them into a comprehensive framework, and discriminant validity issues can be addressed if we consider the existence of an underlying general factor. The fact that we can partial out this component does not mean, however, that we are able to understand it yet, and calling it maladaptation is conjectural. Neither does it mean that we know what exactly maladaptation is. Thus, putting all the pieces together to form a theoretically sound and clinically useful taxonomy of PD still requires extensive empirical work, as well as careful thought.

## Data Availability Statement

The datasets presented in this study can be found in the Open Science Framework [https://osf.io/a3shm/].

## Ethics Statement

This study involves human participants and was reviewed and approved by the Comité de Ética de Investigación con Medicamentos (CEIm, Drug Research Ethics Committee), Hospital Clínic, Barcelona, Spain. Written informed consent to participate in this study was provided by the participants' legal guardian/next of kin.

## Author Contributions

FG, JP, MG, GV, and BS contributed to the conception and design of the study. FG performed the statistical analysis and wrote the first draft of the manuscript. JP, MG, GV, NC, MF, and BS made amendments to the manuscript and rewrote parts of it. All authors contributed to the manuscript revision and approved the submitted version, recruited samples in their respective centers, assessed outpatients, and organized the database.

## Conflict of Interest

The authors declare that the research was conducted in the absence of any commercial or financial relationships that could be construed as a potential conflict of interest.

## References

[1] Huprich SK. Personality disorders in the *ICD-11*: opportunities and challenges for advancing the diagnosis of personality pathology. Curr Psychiatry Rep. (2020) 22:40. 10.1007/s11920-020-01161-4 32519211

[2] Livesley WJ. Why is an evidence-based classification of personality disorder so elusive? Pers Ment Health. (2020) 15:8–25. 10.1002/pmh.1471 32338467

[3] World Health Organization. International Statistical Classification of Diseases for Mortality and Morbidity Statistics. 11th Revision. (2018). Available online at: https://icd.who.int/browse11/l-m/en (accessed March 10, 2021).

[4] American Psychiatric Association. (2013). Diagnostic and Statistical Manual of Mental Disorders. 5th ed. Arlington, VA: American Psychiatric Association.

[5] Mulder RT Newton-Howes G Crawford MJ Tyrer PJ. The central domains of personality pathology in psychiatric patients. J Pers Disord. (2011) 25:364–77. 10.1521/pedi.2011.25.3.364 21699397

[6] Krueger RF Eaton NR Clark LA Watson D Markon KE Derringer J. Deriving an empirical structure of personality pathology for *DSM-5*. J Pers Disord. (2011) 25:170–91. 10.1521/pedi.2011.25.2.170 21466248

[7] Bach B Sellbom M Kongerslev M Simonsen E Krueger RF Mulder R. Deriving *ICD-11* personality disorder domains from *DSM-5* traits: initial attempt to harmonize two diagnostic systems. Acta Psychiatr Scand. (2017) 136:108–17. 10.1111/acps.12748 28504853

[8] Sellbom M Solomon-Krakus S Bach B Bagby RM. Validation of Personality Inventory for *DSM-5* (PID-5) algorithms to assess *ICD-11* personality trait domains in a psychiatric sample. Psychol Assess. (2020) 32:40–9. 10.1037/pas0000746 31204821

[9] Kwapil TR Gross GM Silvia PJ Barrantes-Vidal N. Prediction of psychopathology and functional impairment by positive and negative schizotypy in the Chapmans' Ten-Year Longitudinal Study. J Abn Psychol. (2013) 122:807–15. 10.1037/a0033759 24016018

[10] Oltmanns JR Widiger TA. A self-report measure for the *ICD-11* dimensional trait model proposal: the Personality Inventory for *ICD-11*. Psychol Assess. (2018) 30:154–69. 10.1037/pas0000459 28230410 PMC5930359

[11] Oltmanns JR Widiger TA. The Five-Factor Personality Inventory for *ICD-11*: a facet-level assessment of the *ICD-11* trait model. Psychol Assess. (2020) 32:60–71. 10.1037/pas0000763 31414852 PMC6928398

[12] Aluja A Sayans-Jiménez P García LF Gutiérrez F. Location of *ICD-11* and *DSM-5* dimensional trait models in the Alternative Five Factor personality space. Pers Disord Theory Res Treat. (2020) 12:127–39. 10.1037/per0000460 33630629

[13] Crego C Widiger TA. The convergent, discriminant, and structural relationship of the DAPP-BQ and SNAP with the *ICD-11, DSM-5*, and FFM trait models. Psychol Assess. (2020) 32:18–28. 10.1037/pas0000757 31328932

[14] McCabe GA Widiger TA. A comprehensive comparison of the *ICD-11* and *DSM-5* section III personality disorder models. Psychol Assess. (2020) 32:72–84. 10.1037/pas0000772 31580095

[15] Somma A Gialdi G Fossati A. Reliability and construct validity of the Personality Inventory for *ICD-11* (PiCD) in Italian adult participants. Psychol Assess. (2020) 32:29–39. 10.1037/pas0000766 31414851

[16] Marsh HW Hau K Balla JR Grayson D. Is more ever too much? The number of indicators per factor in confirmatory factor analysis. Multivar Behav Res. (1998) 33:181–220. 10.1207/s15327906mbr3302_1 26771883

[17] Oltmanns JR Smith GT Oltmanns TF Widiger TA. General factors of psychopathology, personality, and personality disorder: across domain comparisons. Clin Psychol Sci. (2018) 6:581–9. 10.1177/2167702617750150 30221082 PMC6132273

[18] Reise SP. The rediscovery of bifactor measurement models. Multivar Behav Res. (2012) 47:667–96. 10.1080/00273171.2012.715555 24049214 PMC3773879

[19] Asparouhov T Muthén B. Exploratory structural equation modeling. Struct Equ Model. (2009) 16:397–438. 10.1080/10705510903008204

[20] Marsh HW Morin AJS Parker PD Kaur G. Exploratory structural equation modeling: an integration of the best features of exploratory and confirmatory factor analysis. Ann Rev Clin Psychol. (2014) 10:85–110. 10.1146/annurev-clinpsy-032813-153700 24313568

[21] Sellbom M Tellegen A. Factor analysis in psychological assessment research: common pitfalls and recommendations. Psychol Assess. (2019) 31:1428–41. 10.1037/pas0000623 31120298

[22] Osborne JW. Effect sizes and the disattenuation of correlation and regression coefficients: lessons from educational psychology. Pract Assess Res Eval. (2003) 8:1–7. 10.7275/0k9h-tq64

[23] Lakens D Scheel AM Isager PM. Equivalence testing for psychological research: a tutorial. Adv Method Pract Psychol Sci. (2018) 1:259–69. 10.1177/2515245918770963

[24] Krueger RF Derringer J Markon KE Watson D Skodol AE. Initial construction of a maladaptive personality trait model and inventory for *DSM-5*. Psychol Med. (2012) 42:1879–90. 10.1017/S0033291711002674 22153017 PMC3413381

[25] Maples JL Carter NT Few LR Crego C Gore WL Samuel DB. Testing whether the *DSM-5* personality disorder trait model can be measured with a reduced set of items: an Item Response Theory investigation of the Personality Inventory for *DSM-5*. Psychol Assess. (2015) 27:1195–210. 10.1037/pas0000120 25844534

[26] Thimm JC Jordan S Bach B. The Personality Inventory for *DSM-5* Short Form (PID-5-SF): psychometric properties and association with Big Five traits and pathological beliefs in a Norwegian population. BMC Psychol. (2016) 4:61. 10.1186/s40359-016-0169-5 27927237 PMC5142430

[27] Gutiérrez F Aluja A Peri JM Calvo N Ferrer M Baillés E. Psychometric properties of the Spanish PID-5 in a clinical and a community sample. Assess. (2017) 24:326–36. 10.1177/1073191115606518 26391204

[28] Gutiérrez F Aluja A Ruiz J García LF Gárriz M Gutiérrez-Zotes A. Personality disorders in the *ICD-11*: Spanish validation of the PiCD and the SASPD in a mixed community and clinical sample. Assess. (2020) 28:759–72. 10.1177/1073191120936357 32583685 PMC7961637

[29] Diaz-Batanero C Ramirez-López J Dominguez-Salas S Fernandez-Calderon F Lozano OM. Personality Inventory for *DSM-5*-Short Form (PID-5-SF): reliability, factorial structure, and relationship with functional impairment in dual diagnosis patients. Assessment. (2019) 26:853–66. 10.1177/1073191117739980 29117705

[30] Osborne JW Fitzpatrick DC. Replication analysis in exploratory factor analysis: what it is and why it makes your analysis better. Pract Assess Res Eval. (2012) 17:1–8. 10.7275/h0bd-4d11

[31] Rosseel Y. lavaan: an R package for structural equation modeling. J Stat Softw. (2012) 48:1–36. 10.18637/jss.v048.i0225601849

[32] Lorenzo-Seva U Ferrando PJ. A general approach for fitting pure exploratory bifactor models. Multivar Behav Res. (2019) 54:15–30. 10.1080/00273171.2018.1484339 30160535

[33] Ferrando PJ Lorenzo-Seva U. Program FACTOR at 10: origins, development and future directions. Psicothema. (2017) 29:236–40. 10.7334/psicothema2016.304 28438248

[34] Reddy MS Starlin MV Reddy S. Obsessive-compulsive (anankastic) personality disorder: A poorly researched landscape with significant clinical relevance. Indian J Psychol Med. (2016) 38:1. 10.4103/0253-7176.175085 27011394 PMC4782437

[35] Caspi A Moffitt TE. All for one and one for all: mental disorders in one dimension. Am J Psychiatry. (2018) 175:831–44. 10.1176/appi.ajp.2018.17121383 29621902 PMC6120790

[36] Watson D Clark LA. Personality traits as an organizing framework for personality pathology. Pers Ment Health. (2020) 14:51–75. 10.1002/pmh.1458 31309725 PMC6960378

[37] Bastiaens T Smits D De Hert M Thys E Bryon H Sweers K. The relationship between the Personality Inventory for the *DSM-5* (PID-5) and the psychotic disorder in a clinical sample. Assessment. (2019) 26:315–23. 10.1177/1073191117693922 29214869

[38] Heath LM Drvaric L Hendershot CS Quilty LC Bagby RM. Normative and maladaptive personality trait models of mood, psychotic, and substance use disorders. J Psychopathol Behav Assess. (2018) 40:606–13. 10.1007/s10862-018-9688-0 30459484 PMC6223804

[39] Longenecker JM Krueger RF Sponheim SR. Personality traits across the psychosis spectrum: a hierarchical taxonomy of psychopathology conceptualization of clinical symptomatology. Pers Ment Health. (2020) 14:88–105. 10.1002/pmh.1448 31309736 PMC6960376

[40] Morey LC Good EW Hopwood CJ. Global personality dysfunction and the relationship of pathological and normal trait domains in the *DSM-5* Alternative Model for personality disorders. J Pers. (2020). 10.1111/jopy.12560. [Epub ahead of print]. 32422689

[41] Anderson JL Sellbom M. Evaluating the *DSM-5* Section III personality disorder impairment criteria. Pers Disord Theory Res Treat. (2018) 9:51–61. 10.1037/per0000217 27618341

[42] Sleep C Lynam DR Miller JD. Personality impairment in the *DSM-5* and *ICD-11*: current standing and limitations. Curr Opin Psychiatry. (2021) 34:39–43. 10.1097/YCO.0000000000000657 33252428

