# Supplementary material for: Integration of the ICD-11 and DSM-5 Dimensional Systems for Personality Disorders Into a Unified Taxonomy With Non-overlapping Traits
Source: Front Psychiatry. 2021 Apr 6;12:591934. doi: 10.3389/fpsyt.2021.591934 (PMC8055818; doi:10.3389/fpsyt.2021.591934)

Supplementary Material

Integration of the ICD-11 and DSM-5 dimensional systems for personality disorders into a unified taxonomy with non-overlapping traits

Supplementary Table 1. Summary Statistics for the PiCD and the PID-5 (n = 677).

	Raw score		T-score		Cronbach's alpha
	Mean	(SD)	Mean	(SD)	
PiCD Neg. Affectivity	41.55	(8.84)	61.8	(10.9)	.84
PiCD Detachment	29.80	(9.11)	55.7	(12.0)	.83
PiCD Dissociality	23.80	(7.85)	53.5	(11.9)	.80
PiCD Disinhibition	28.69	(9.04)	56.3	(12.2)	.84
PiCD Anankastia	39.23	(7.96)	49.5	(11.2)	.78
PiCD Total	163.08	(24.96)	59.7	(11.9)	.83
PID-5 Emotional Lability	1.72	(.82)	58.0	(11.6)	.78
PID-5 Anxiousness	1.90	(.81)	60.7	(11.4)	.80
PID-5 Separat. Insecurity	1.35	(.88)	52.5	(11.4)	.82
PID-5 Hostility	1.32	(.83)	57.4	(12.8)	.82
PID-5 Perseveration	1.49	(.75)	57.6	(12.9)	.77
PID-5 Submissiveness	1.10	(.74)	55.4	(12.3)	.84
PID-5 Restricted Affect	1.08	(.77)	54.6	(12.7)	.76
PID-5 Withdrawal	1.15	(.80)	59.8	(13.8)	.82
PID-5 Anhedonia	1.43	(.90)	67.0	(16.5)	.86
PID-5 Depressiveness	1.06	(.90)	62.6	(18.3)	.85
PID-5 Intim. Avoidance	.86	(.87)	54.9	(14.4)	.87
PID-5 Suspiciousness	1.10	(.73)	59.2	(14.2)	.71
PID-5 Manipulativeness	.69	(.73)	53.7	(14.2)	.83
PID-5 Deceitfulness	.66	(.71)	54.7	(13.9)	.78
PID-5 Grandiosity	.46	(.59)	49.6	(11.3)	.74
PID-5 Attention Seeking	.78	(.80)	50.5	(11.6)	.88
PID-5 Callousness	.39	(.59)	52.5	(13.9)	.82
PID-5 Irresponsibility	.83	(.69)	58.0	(14.9)	.67
PID-5 Impulsivity	1.28	(.89)	54.7	(12.9)	.90
PID-5 Distractibility	1.73	(.87)	60.9	(12.1)	.88
PID-5 Risk Taking	.74	(.76)	52.8	(13.1)	.85
PID-5 Rigid Perfectionism	1.42	(.83)	53.8	(13.1)	.81
PID-5 Unusual Beliefs	.73	(.75)	57.1	(15.1)	.71
PID-5 Eccentricity	1.26	(.93)	61.5	(15.0)	.88
PID-5 Perceptive Distortion	.52	(.65)	57.8	(17.5)	.72
PID-5 Neg. Affectivity	1.41	(.57)	60.7	(13.1)	.93
PID-5 Detachment	1.13	(.62)	61.9	(14.4)	.89
PID-5 Antagonism	.60	(.53)	52.7	(12.9)	.92
PID-5 Disinhibition	1.14	(.60)	59.0	(13.3)	.89
PID-5 Psychoticism	.84	(.66)	61.2	(16.3)	.88
PID-5 Total	1.08	(.45)	61.8	(13.8)	.96

Note. PID-5 = Personality Inventory for the DSM-5 – Short Form, PiCD = Personality Inventory for ICD-11. T-scores are based on the respective Spanish validation studies (27,28)

Supplementary Table 2. Multiple Regression Coefficients for the PID-5 Predicting the PiCD and vice versa (n = 677).

	R	Adj R^2	Standardized betas (domains)	R	Adj R^2	Standardized betas (facets)
PiCD Neg. Affectivity	.78	.61	Negative Affectivity (.816), Antagonism (-.107)	.80	.64	Anxiousness (.366), Emotional Lability (.184), Depressiveness (.133), Impulsivity (.102), Rigid Perfectionism (.101)
PiCD Detachment	.72	.52	Detachment (.724)	.80	.63	Withdrawal (.403), Restricted Affect (.368), Attention Seeking (-.105)
PiCD Dissociality	.76	.58	Antagonism (.624), Psychoticism (.133), Disinhibition (.113)	.80	.63	Manipulativeness (.370), Hostility (.170), Callousness (.164), Risk Taking (.115), Unusual Beliefs (.113), Attention Seeking (.100)
PiCD Disinhibition	.80	.65	Disinhibition (.804)	.85	.71	Impulsivity (.385), Irresponsibility (.325), Rigid Perfectionism (-.190), Distractibility (.125), Risk Taking (.116), Depressivity (.106)
PiCD Anankastia	.55	.30	Disinhibition (-.746), Negative Affectivity (.441)	.70	.48	Rigid Perfectionism (.442), Impulsivity (-.408), Risk Taking (-.166), Anxiousness (.125), Depressivity (-.125), Restricted Affect (.118), Grandiosity (.102)
PID-5 Neg. Affectivity	.81	.65	Negative Affectivity (.651), Dissociality (.157), Disinhibition (.121)			
PID-5 Detachment	.75	.56	Detachment (.656), Disinhibition (.154)			
PID-5 Antagonism	.75	.56	Dissociality (.681), Disinhibition (.149)			
PID-5 Disinhibition	.84	.70	Disinhibition (.565), Dissociality (.164), Negative Affectivity (.174), Anankastia (-.134)			
PID-5 Psychoticism	.62	.38	Negative Affectivity (.255), Dissociality (.234), Detachment (.225), Disinhibition (.164)			

Note. PID-5 = Personality Inventory for the DSM-5 – Short Form, PiCD = Personality Inventory for ICD-11. Only betas > .100 are shown.

Supplementary Table 3. Equivalence Between the PiCD and PID-5 Four Corresponding Domains Using the Paired TOST Procedure (n = 677).

	Mean PID-5	Mean PiCD	ΔT	t	p
**Negative Affectivity**					
Total	**60.7**	**61.8**	**-1.13**	**12.00**	**<.001**
Severity < 50	44.0	48.8	-4.76	0.30	.383
Severity = 50-60	**55.8**	**58.7**	**-2.97**	**3.71**	**<.001**
Severity = 60-70	**64.7**	**65.6**	**-0.87**	**8.03**	**<.001**
Severity > 70	73.9	64.5	9.38	5.07	1.00
**Detachment**					
Total	61.9	55.8	6.14	2.93	.998
Severity < 50	**47.7**	**45.4**	**2.27**	**-3.30**	**<.001**
Severity = 50-60	57.3	52.0	5.31	0.48	.685
Severity = 60-70	66.3	59.4	6.92	2.62	.995
Severity > 70	**64.2**	**65.2**	**-0.93**	**4.21**	**<.001**
**Dissociality /Antagonism**					
Total	**52.7**	**53.5**	**-0.80**	**11.81**	**<.001**
Severity < 50	**43.9**	**44.1**	**-0.22**	**8.25**	**<.001**
Severity = 50-60	**48.2**	**49.0**	**-0.81**	**7.46**	**<.001**
Severity = 60-70	**53.8**	**54.9**	**-1.06**	**5.78**	**<.001**
Severity > 70	73.1	67.8	5.21	0.28	.610
**Disinhibition**					
Total	**59.0**	**56.3**	**2.74**	**-7.33**	**<.001**
Severity < 50	**45.7**	**45.4**	**0.37**	**-7.67**	**<.001**
Severity = 50-60	**54.0**	**52.3**	**1.75**	**-6.45**	**<.001**
Severity = 60-70	**61.5**	**58.2**	**3.33**	**-2.94**	**.002**
Severity > 70	**75.6**	**71.8**	**3.89**	**-1.79**	**.038**

Note. PID-5 = Personality Inventory for the DSM-5 – Short Form, PiCD = Personality Inventory for ICD-11, TOST = Two One-Sided Test. T-scores are based on the respective Spanish validation studies (26,27). Statistically significant equivalences are in bold type.

Supplementary Table 4. Domain-Level 4-Factor Solution for the PiCD and the PID-5 (n = 677).

	**Negative Affectivity – Psychoticism**	**Disso-ciality**	**Detach-ment**	**Disinhi-bition**
Factor solution				
PID-5 Neg. Affectivity	**1.04**	-.01	-.03	-.09
PiCD Neg. Affectivity	**.85**	-.16	.00	.01
PID-5 Psychoticism	**.47**	.13	.20	.10
PID-5 Antagonism	-.01	**.97**	-.06	-.01
PiCD Dissociality	.04	**.72**	.07	.06
PiCD Detachment	-.04	-.02	**.98**	-.05
PID-5 Detachment	.21	.00	**.67**	.05
PiCD Anankastia	.04	.14	.20	**-.75**
PiCD Disinhibition	**.32**	.02	.04	**.73**
PID-5 Disinhibition	**.47**	.09	.02	**.60**
Correlations with domains				
PiCD Neg. Affectivity	**.78**	.28	**.35**	.17
PiCD Detachment	**.37**	.20	**.99**	.18
PiCD Dissociality	**.45**	**.83**	**.30**	**.45**
PiCD Disinhibition	**.54**	**.55**	**.37**	**.89**
PiCD Anankastia	.01	-.14	.08	**-.67**
PID-5 Neg. Affectivity	**1.00**	**.47**	**.42**	.18
PID-5 Detachment	**.51**	**.31**	**.81**	.28
PID-5 Antagonism	**.43**	**.98**	.19	**.44**
PID-5 Disinhibition	**.68**	**.63**	**.41**	**.82**
PID-5 Psychoticism	**.65**	**.48**	**.48**	**.35**
Intercorrelations				
Neg. Affectivity-Psychoticism	—			
Dissociality	**.51**	—		
Detachment	**.46**	.28	—	
Disinhibition	.27	**.51**	.26	—

Note. PID-5 = Personality Inventory for the DSM-5 – Short Form, PiCD = Personality Inventory for ICD-11. Loadings and correlations ≥ .30 are in bold type.

Supplementary Table 5. Domain-Level 5-Factor Solution for the PiCD and the PID-5 (n = 677).

	**Negative Affectivity – Psychoticism**	**Disinhi-bition**	**Disso-ciality**	**Detach-ment**	**F5**
Factor solution					
PID-5 Neg. Affectivity	**.97**	-.05	.01	-.01	.04
PiCD Neg. Affectivity	**.76**	.01	-.04	-.03	**.44**
PID-5 Psychoticism	**.49**	.09	.13	.20	-.10
PiCD Anankastia	.26	**-.80**	.08	.16	.03
PiCD Disinhibition	.03	**.79**	.18	.05	.23
PID-5 Disinhibition	**.32**	**.62**	.15	.03	.00
PiCD Dissociality	-.03	.00	**.88**	.04	.05
PID-5 Antagonism	.05	.02	**.83**	-.08	-.10
PiCD Detachment	-.02	-.04	.01	**.96**	.08
PID-5 Detachment	.25	.06	-.03	**.68**	-.10
Correlations with domains					
PiCD Neg. Affectivity	**.82**	.28	**.33**	**.30**	**.71**
PiCD Detachment	**.38**	.22	.25	**.99**	.14
PiCD Dissociality	**.45**	**.43**	**.94**	.28	-.05
PiCD Disinhibition	**.53**	**.93**	**.60**	.35	.16
PiCD Anankastia	.03	**-.65**	-.13	.07	.21
PID-5 Neg. Affectivity	**.98**	**.40**	**.51**	.40	.22
PID-5 Detachment	**.54**	**.37**	**.34**	**.82**	-.08
PID-5 Antagonism	**.44**	**.45**	**.91**	.17	-.23
PID-5 Disinhibition	**.70**	**.90**	**.66**	**.39**	-.07
PID-5 Psychoticism	**.69**	**.46**	**.52**	**.48**	-.07
Intercorrelations					
Neg. Affectivity– Psychoticism	—				
Disinhibition	**.47**	—			
Dissociality	**.56**	**.55**	—		
Detachment	**.44**	**.30**	**.30**	—	
F5	.24	-.10	-.13	.06	—

Note. PID-5 = Personality Inventory for the DSM-5 – Short Form, PiCD = Personality Inventory for ICD-11. Loadings and correlations ≥ .30 are in bold type.

Supplementary Table 6. ESEM 5-Factor Solution for the PiCD Domains and PID-5 Facets Targeted to the EFA Solution (n = 677).

	**Neg. Affectivity**	**Detach-ment**	**Disso-ciality**	**Disinhi-bition**	**Psycho-ticism**
Factor Solution					
PiCD Negative Affectivity	**.87**	-.09	-.05	-.10	.01
PiCD Detachment	.25	**.74**	.02	-.02	.03
PiCD Dissociality	.04	.02	**.74**	.02	.12
PiCD Disinhibition	**.42**	.02	.24	**.59**	.02
PiCD Anankastia	.03	.12	.03	**-.77**	-.03
PID-5 Anxiousness	**.81**	-.07	-.02	-.25	-.02
PID-5 Emotional Lability	**.72**	**-.31**	-.11	.04	.12
PID-5 Perseveration	**.67**	.00	.11	.03	-.04
PID-5 Anhedonia	**.64**	**.37**	.05	.03	-.07
PID-5 Depressiveness	**.64**	.26	-.05	.08	.11
PID-5 Distractibility	**.59**	.09	-.03	.23	-.03
PID-5 Separation Insecurity	**.56**	-.27	.06	-.05	.01
PID-5 Hostility	**.53**	.06	.23	.07	.01
PID-5 Submissiveness	**.52**	.07	.02	-.08	-.14
PID-5 Impulsivity	**.47**	-.16	.15	**.45**	.13
PID-5 Suspiciousness	**.43**	.07	.23	-.12	.26
PID-5 Restricted Affect	-.04	**.71**	.23	-.02	.02
PID-5 Withdrawal	**.36**	**.62**	-.04	-.06	.05
PID-5 Intimacy Avoidance	.04	**.45**	-.05	.16	.15
PID-5 Manipulativeness	-.10	-.07	**.88**	-.02	.01
PID-5 Deceitfulness	.07	-.01	**.81**	.05	-.05
PID-5 Attention Seeking	.09	**-.32**	**.65**	-.02	-.01
PID-5 Grandiosity	.01	.00	**.63**	-.21	.04
PID-5 Callousness	-.05	.21	**.59**	.10	.10
PID-5 Irresponsibility	.27	.15	**.41**	**.40**	-.06
PID-5 Rigid Perfectionism	**.40**	.00	.20	**-.51**	.15
PID-5 Unusual Beliefs	.02	.01	.07	.01	**.81**
PID-5 Perceptive Distortion	.14	.03	-.04	-.03	**.72**
PID-5 Eccentricity	**.33**	.16	.07	.02	**.39**
PID-5 Risk Taking	-.02	-.03	**.37**	.26	**.38**
Correlations with domains					
PiCD Neg. Affectivity	**.86**	.06	.25	-.04	**.41**
PiCD Detachment	**.41**	**.86**	.21	.04	**.36**
PiCD Dissociality	**.38**	.15	**.84**	.22	**.47**
PiCD Disinhibition	**.59**	.17	**.56**	**.75**	**.43**
PiCD Anankastia	-.02	.10	-.14	**-.83**	-.07
PID-5 Neg. Affectivity	**.95**	.15	**.44**	- .03	**.58**
PID-5 Detachment	**.54**	**.85**	**.31**	.14	**.46**
PID-5 Antagonism	**.35**	.04	**.96**	.20	**.40**
PID-5 Disinhibition	**.69**	.19	**.63**	**.62**	**.58**
PID-5 Psychoticism	**.62**	**.34**	**.44**	.13	**.95**

Intercorrelations					
Neg. Affectivity	—				
Detachment	.21	—			
Dissociality	**.40**	.13	—		
Disinhitibition	.11	.05	.27	—	
Psychoticism	**.56**	.29	**.46**	.14	—

Note. PID-5 = Personality Inventory for the DSM-5 – Short Form, PiCD = Personality Inventory for ICD-11. Correlations and factor loadings ≥ .30 are in bold type.

Supplementary Table 7. ESEM 4-Factor Solution for the PiCD Domains and PID-5 Facets Targeted to the EFA Solution (n = 677).

	**Negative Affectivity – Psychoticism**	**Detachment**	**Dissociality**	**Dishinhi-bition**
Factor Solution				
PiCD Negative affectivity	**.88**	-.10	-.04	-.05
PiCD Detachment	.25	**.75**	.01	-.02
PiCD Dissociality	.03	.02	**.80**	.02
PiCD Disinhibition	.23	.03	.27	**.64**
PiCD Anankastia	.24	.09	.00	**-.80**
PID-5 Anxiousness	**.85**	-.09	-.02	-.20
PID-5 Emotional Lability	**.74**	-.29	-.06	.09
PID-5 Depressiveness	**.64**	.27	-.02	.12
PID-5 Perseveration	**.62**	-.02	.11	.07
PID-5 Rigid Perfectionism	**.59**	.00	.25	**-.51**
PID-5 Anhedonia	**.58**	**.35**	.03	.07
PID-5 Separation Insecurity	**.56**	-.28	.08	-.01
PID-5 Suspiciousness	**.54**	.11	**.32**	-.09
PID-5 Distractibility	**.49**	.09	-.03	.28
PID-5 Hostility	**.48**	.05	.24	.10
PID-5 Submissiveness	**.47**	.03	-.02	-.06
PID-5 Eccentricity	**.46**	.23	.20	.05
PID-5 Perceptive Distortion	**.42**	.16	.17	.00
PID-5 Impulsivity	**.37**	-.13	.21	**.50**
PID-5 Unusual Beliefs	**.32**	.15	**.30**	.04
PID-5 Manipulativeness	-.14	-.10	**.90**	-.03
PID-5 Deceitfulness	-.02	-.04	**.81**	.06
PID-5 Attention Seeking	.05	**-.34**	**.68**	-.01
PID-5 Grandiosity	.05	-.02	**.66**	-.22
PID-5 Callousness	-.08	.22	**.63**	.11
PID-5 Risk Taking	.04	.03	**.50**	.28
PID-5 Restricted Affect	-.04	**.71**	.23	-.03
PID-5 Withdrawal	**.38**	**.63**	-.04	-.04
PID-5 Intimacy Avoidance	.05	**.48**	-.01	.16
PID-5 Irresponsibility	.10	.14	**.41**	**.42**
Correlations with domains				
PiCD Neg. Affectivity	**.86**	.07	.29	.20
PiCD Detachment	**.42**	**.86**	.25	.13
PiCD Dissociality	**.37**	.18	**.85**	.29
PiCD Disinhibition	**.55**	.20	**.58**	**.85**
PiCD Anankastia	.03	.08	-.13	**-.78**
PID-5 Neg. Affectivity	**.96**	.18	**.50**	.24
PID-5 Detachment	**.54**	**.86**	**.35**	.26
PID-5 Antagonism	**.32**	.07	**.95**	.27
PID-5 Disinhibition	**.66**	.22	**.66**	**.76**
PID-5 Psychoticism	**.66**	**.40**	**.53**	**.30**

Intercorrelations				
Neg. Affectivity - Psychoticism	—			
Detachment	.24	—		
Dissociality	**.43**	.20	—	
Disinhibition	**.32**	.10	**.35**	—

Note. PID-5 = Personality Inventory for the DSM-5 – Short Form, PiCD = Personality Inventory for ICD-11. Correlations and factor loadings ≥ .30 are in bold type.

Supplementary Table 8. ESEM 4+1-Factor Solution for the PiCD Domains and PID-5 Facets Targeted to Purely Exploratory Bifactor Analysis (n = 677).

	**g-PD**	**Negative Affectivity**	**Detachment**	**Dissociality**	**Dishinhi-bition**
Factor Solution					
PiCD Neg. Affectivity	**.45**	**.72**	-.02	-.08	.15
PiCD Detachment	**.52**	.15	**.64**	-.18	-.08
PiCD Dissociality	**.55**	-.03	.05	**.60**	.10
PiCD Disinhibition	**.61**	.07	-.05	.10	**.63**
PiCD Anankastia	-.19	**.33**	.27	.07	**-.65**
PID-5 Anxiousness	**.36**	**.72**	.04	-.05	.03
PID-5 Emotional Lability	**.37**	**.56**	-.26	-.08	.23
PID-5 Perseveration	**.45**	**.50**	.06	.03	.24
PID-5 Rigid Perfectionism	**.34**	**.49**	.10	.17	**-.32**
PID-5 Separation Insecurity	.24	**.48**	-.17	.09	.17
PID-5 Anhedonia	**.55**	**.46**	**.37**	-.12	.19
PID-5 Submissiveness	.19	**.46**	.14	-.02	.11
PID-5 Depressiveness	**.62**	**.44**	.20	-.18	.17
PID-5 Distractibility	**.44**	**.37**	.09	-.12	**.35**
PID-5 Hostility	**.51**	**.36**	.08	.12	.23
PID-5 Suspiciousness	**.63**	**.35**	.06	.14	-.02
PID-5 Restricted Affect	**.45**	-.08	**.61**	.03	-.10
PID-5 Withdrawal	**.52**	.26	**.53**	-.22	-.06
PID-5 Intimacy Avoidance	**.40**	-.08	**.30**	-.17	.01
PID-5 Manipulativeness	**.39**	-.11	.02	**.73**	.13
PID-5 Deceitfulness	**.44**	.01	.09	**.65**	.23
PID-5 Attention Seeking	.24	.07	-.18	**.62**	.17
PID-5 Grandiosity	**.32**	.06	.10	**.55**	-.08
PID-5 Callousness	**.50**	-.12	.19	**.43**	.12
PID-5 Irresponsibility	**.52**	.04	.13	.25	**.46**
PID-5 Impulsivity	**.56**	.18	-.21	.09	**.50**
PID-5 Unusual Beliefs	**.80**	-.04	-.20	.03	-.18
PID-5 Perceptive Distortion	**.72**	.10	-.13	-.06	-.17
PID-5 Eccentricity	**.70**	.21	.05	-.02	-.02
PID-5 Risk Taking	**.62**	-.16	-.15	.28	.17
Correlations with domains					
PiCD Neg. Affectivity	**.47**	**.76**	-.12	.03	.10
PiCD Detachment	**.55**	.04	**.68**	-.19	-.03
PiCD Dissociality	**.58**	.05	.07	**.64**	.15
PiCD Disinhibition	**.64**	.04	.00	.14	**.68**
PiCD Anankastia	-.19	**.39**	.17	.11	**-.72**
PID-5 Neg. Affectivity	**.65**	**.75**	-.08	.15	.07
PID-5 Detachment	**.67**	.09	**.64**	-.15	.07
PID-5 Antagonism	**.50**	.08	.06	**.84**	.19
PID-5 Disinhibition	**.75**	.14	-.04	.20	**.52**
PID-5 Psychoticism	**.91**	.15	-.15	.00	-.18

Intercorrelations					
g-PD	--				
Neg. Affectivity - Psychoticism	.06	--			
Detachment	.05	-.18	--		
Dissociality	.05	.12	-.04	--	
Disinhitibition	.05	-.11	.07	.04	--

Note. PID-5 = Personality Inventory for the DSM-5 – Short Form, PiCD = Personality Inventory for ICD-11. Correlations and factor loadings ≥ .30 are in bold type.

Supplementary Figure 1. Parallel Analysis Scree Plots for the Domain-Level (a) and Facet-Level (b) Analyses (n = 677).

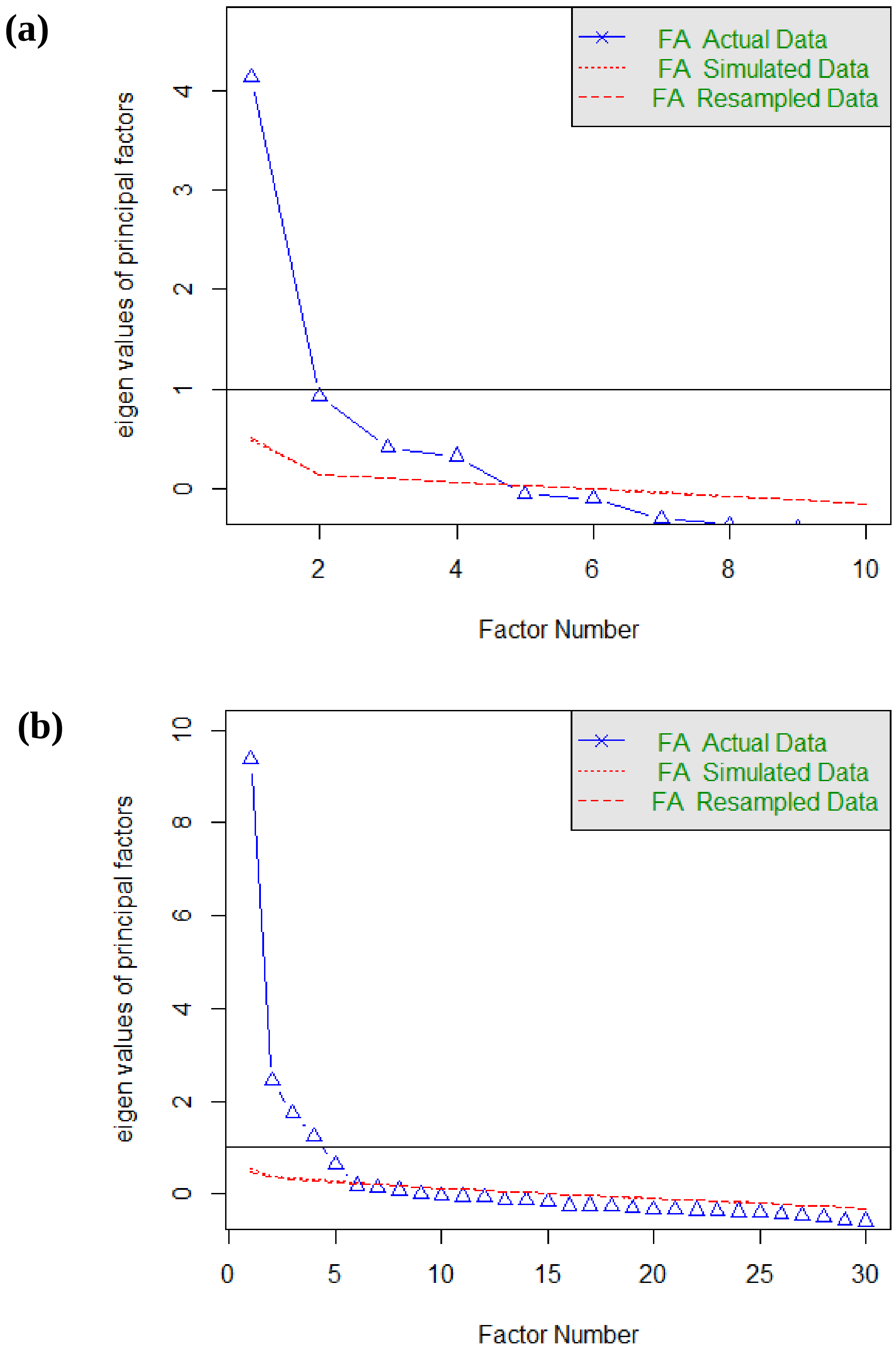

Supplement: Supplementary file 1 [file Data_Sheet_1.pdf]
